# An integrated model incorporating deep learning, hand-crafted radiomics and clinical and US features to diagnose central lymph node metastasis in patients with papillary thyroid cancer

**DOI:** 10.1186/s12885-024-11838-1

**Published:** 2024-01-12

**Authors:** Yang Gao, Weizhen Wang, Yuan Yang, Ziting Xu, Yue Lin, Ting Lang, Shangtong Lei, Yisheng Xiao, Wei Yang, Weijun Huang, Yingjia Li

**Affiliations:** 1 Department of Ultrasound, Nanfang Hospital, Southern Medical University, 1838 Guangzhou Avenue North, Baiyun District, Guangzhou, Guangdong Province P. R. China; 2 Guangdong Provincial Key Laboratory of Medical Image Processing, School of Biomedical Engineering, Southern Medical University, 1838 Guangzhou Avenue North, Baiyun District, Guangzhou, Guangdong Province P. R. China; 3 Department of Ultrasound, the First People’s Hospital of Foshan, Lingnan Avenue North No.81, Foshan, Guangdong Province P. R. China; 4 Department of General Surgery, Nanfang Hospital, Southern Medical University, Guangzhou, P. R. China

**Keywords:** Ultrasonography, Papillary thyroid carcinoma, Lymph node metastasis, Deep learning

## Abstract

**Objective:**

To evaluate the value of an integrated model incorporating deep learning (DL), hand-crafted radiomics and clinical and US imaging features for diagnosing central lymph node metastasis (CLNM) in patients with papillary thyroid cancer (PTC).

**Methods:**

This retrospective study reviewed 613 patients with clinicopathologically confirmed PTC from two institutions. The DL model and hand-crafted radiomics model were developed using primary lesion images and then integrated with clinical and US features selected by multivariate analysis to generate an integrated model. The performance was compared with junior and senior radiologists on the independent test set. SHapley Additive exPlanations (SHAP) plot and Gradient-weighted Class Activation Mapping (Grad-CAM) were used for the visualized explanation of the model.

**Results:**

The integrated model yielded the best performance with an AUC of 0.841. surpassing that of the hand-crafted radiomics model (0.706, *p* < 0.001) and the DL model (0.819, *p* = 0.26). Compared to junior and senior radiologists, the integrated model reduced the missed CLNM rate from 57.89% and 44.74–27.63%, and decreased the rate of unnecessary central lymph node dissection (CLND) from 29.87% and 27.27–18.18%, respectively. SHAP analysis revealed that the DL features played a primary role in the diagnosis of CLNM, while clinical and US features (such as extrathyroidal extension, tumour size, age, gender, and multifocality) provided additional support. Grad-CAM indicated that the model exhibited a stronger focus on thyroid capsule in patients with CLNM.

**Conclusion:**

Integrated model can effectively decrease the incidence of missed CLNM and unnecessary CLND. The application of the integrated model can help improve the acceptance of AI-assisted US diagnosis among radiologists.

**Supplementary Information:**

The online version contains supplementary material available at 10.1186/s12885-024-11838-1.

## Introduction

In recent years, the incidence of thyroid cancer has increased significantly worldwide, with papillary thyroid carcinoma (PTC) accounting for most cases [1]. PTC is characterized by early metastasis to cervical lymph nodes (LNs), particularly in the central region [2]. The reported rate of central LN metastasis (CLNM) in patients is approximately 50% [3], which is a known risk factor for recurrence and adversely affects overall survival [4, 5]. The necessity of prophylactic central LN dissection (pCLND) remains a subject of debate in thyroid cancer treatment. In China, the latest guidelines recommend routine pCLND at least ipsilateral to the lesion [6]. While pCLND can effectively reduce the need for reoperation in cases of recurrence, it also leads to unnecessary CLND procedures. US is the most commonly used method for preoperative LN assessment in PTC [7]. However, its sensitivity in identifying CLNM ranges from only 26–47%, which is insufficient for accurate assessment [8]. Hence, a more sensitive preoperative assessment of CLNM is crucial for patients with PTC to reduce unnecessary CLND.

Radiomics represents a high-throughput data mining approach for the discovery of novel imaging biomarkers and uses two main approaches: hand-crafted radiomics and deep learning [9]. In recent years, both hand-crafted radiomics and deep learning, have shown powerful analytical capabilities in extracting intricate and multi-layered features from medical images [10, 11]. Hand-crafted radiomics focuses on the mathematical manipulation of images to produce traditional features of texture and shape, etc. whereas the DL approaches can generate high-dimensional features to represent the deep image information of the tumour through end-to-end learning [12]. We previously reported a preliminary small sample study of CLNM using hand-crafted radiomics, which acquired good performance [13]. To date, most studies have independently employed DL and hand-crafted radiomics features, and far fewer studies have attempted to fuse these two features from US images. It is worth noting that features extracted by DL models may be sensitive to global translation, rotation, and scaling while hand-crafted radiomics features such as intensity features are not [14, 15], Therefore, we hypothesize that hand-crafted radiomics features and DL features extracted from US images could be complementary, and their combination may yield improved prediction outcomes.

However, unlike radiologists who incorporate clinical and US information to make diagnoses, most AI models only provide output results without revealing their decision-making process. This lack of transparency is considered one of the reasons why radiologists are skeptical about the clinical application of AI models. Previous studies have highlighted the significance of clinical and US characteristics (e.g., age, gender, and tumour size) in distinguishing CLNM [16]. Nevertheless, the lack of information such as age and gender in the images, and data pre-processing such as resizing and normalisation, makes detecting these information challenging in machine learning [17]. By integrating clinical and US features into AI models, it may be possible to improve the predictive efficacy of the models as well as the acceptance from radiologists.

Hence, this study aimed to develop and validate whether an integrated model incorporating DL, hand-crafted radiomics and clinical and US features can improve the performance to diagnose CLNM in patients with PTC, in order to reduce the miss rate of CLNM, unnecessary CLND and improve the acceptance of AI-assisted US diagnosis for radiologists.

## Patients and methods

### Patients

The Ethics Committees of Nanfang Hospital of South Medical University and the First People’s Hospital of Foshan (NFEC-202,008-K6) approved this retrospective study. The requirement for informed consent was waived. The checklist for Artificial Intelligence in Medical Imaging (CLAIM) and EvaluAtion of Radiomics research (CLEAR) were applied as step-by-step reporting guideline for this study, which is presented in a Supplementary Material 1 and 2 [18, 19]. The inclusion and exclusion criteria were as follows:

### Inclusion criteria

Patients were enrolled if they satisfied all the following inclusion criteria: (1) were confirmed to have PTC after lobectomy or total thyroidectomy; (2) underwent CLND with a pathological examination; (3) the thyroid US examination was performed at our hospital within one month before the operation.

### Exclusion criteria

(1) had other malignancies or distant metastases at diagnosis; (2) received preoperative head and neck therapies such as radiotherapy, chemotherapy, or radiofrequency ablation; (3) with missing data; (4) with poor image quality.

**Fig. 1:**
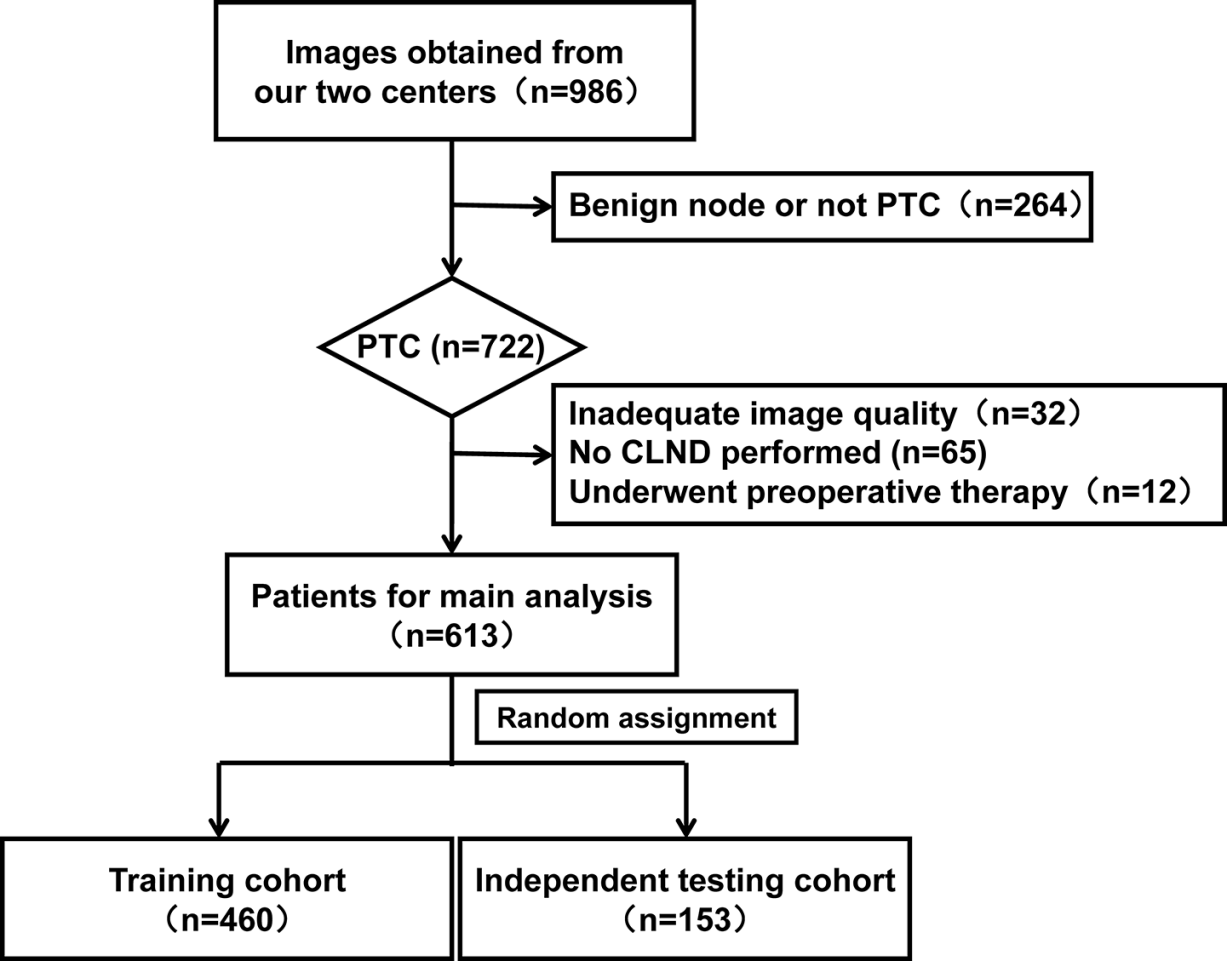
Flow Chart of participants recruitment. CLND, central lymph node dissection; PTC, papillary thyroid carcinoma

After undergoing a rigorous inclusion and exclusion process, datasets of 613 patients treated in our clinical centres from March 2019 to July 2020 were included. The participant recruitment flow is shown in Fig. 1. The participants were randomly divided into training and independent test cohorts for further analysis.

### Acquisition and selection of clinical and ultrasound features

The choice of US machine was not limited, and most data was obtained using devices such as Siemens Sequoia, Supersonic Aixplorer, and Toshiba Aplio 500, stored in the DICOM format. The risk factors for CLNM were identified by the following variables: gender, age, and US features of thyroid tumours following the C-TIRADS and ATA guidelines [7, 20]. These features encompassed tumour size, hypoechoic solid composition, multifocality, aspect ratio, posterior acoustic attenuation, tumour location, extrathyroidal extension (ETE), acoustic halo, microcalcification, and the internal tumour vascularity. Age was dichotomized at 55 years following the 8th American Joint Commission on Cancer staging system. In cases with multifocality, the largest nodule was chosen as the representative. The tumour vascularity was graded from 0 to 3 by colour Doppler flow imaging (CDFI) following the Adler standard [21]. The US features were re-evaluated by two radiologists with four and seven years of experience in thyroid US diagnosis. Both radiologists were blinded to clinical information and pathological diagnosis. The agreement between them was assessed, and in case of any disagreement, a senior radiologist with over 20 years of experience made the final decision. Subsequently, multivariate logistic regression analysis and likelihood ratio tests for positive selection were used in the training cohort to screen for the above mentioned clinical and US features that can effectively differentiate the presence of CLNM.

### Evaluation of lymph node metastases by radiologists

The preoperative examination of LNs was conducted on all patients by a team of five radiologists, comprising two senior radiologists with 15 and 17 years of experience, and three junior radiologists with 3, 5, and 6 years of experience, respectively. The diagnostic accuracy of the two groups of radiologists, differing in seniority, was determined by comparing the LNs status reported by US with the corresponding postoperative pathological results. Based on the ACR TI-RADS [22], LNs exhibiting one or more suspicious US features (roundness, loss of the normal echogenic hilum, internal microcalcifications, cystic changes, hyperechogenicity, or presence of peripheral flow) were classified as US-reported CLNM.

### Region of interest segmentation and development of the hand-crafted radiomics model

The manual segmentation of regions of interest (ROI) was independently performed using ITK-SNAP (version 3.8) by a radiologist with five years of experience, followed by another radiologist with seven years of experience reviewing the ROI and reaching a consensus. Before formally drawing ROIs, we randomly selected 30 images for consistency analysis, and the two radiologists have excellent consistency, with a dice coefficient of 0.946.

Radiomic features were extracted using the open-source Python package “pyradiomics” (version 3.1.0) [23]. A total of 783 features including 18 first-order statistics, 68 texture features, 9 shape features, 344 wavelet decompositions, and 344 Laplacian of Gaussian features were extracted from the US images by the delineated ROI. The definitions of each feature group are listed in Supplementary Material 3 S1. The least absolute shrinkage and selection operator (LASSO) logistic regression analysis method was employed to select the radiomics feature on the training dataset. The selected radiomics features are listed in Supplementary Material 3 S2. We followed a support vector machine (SVM) to establish the prediction model, with the regularization parameter and kernel type tuning conducted by 10-fold cross-validation in the training set. The LASSO and SVM were performed by the “scikit-learn” package (version 0.24.2).

### Development of the deep learning model

A convolution neural network (CNN) was built to utilize deep features of US images to predict central lymph node metastasis. The US images of the patient in the training cohort were randomly divided into training and validation datasets with a ratio of 2:1. These images were cropped based on the delineated ROIs, resized to 224 × 224, and then normalized the grayscale to [0, 1] in the pre-processing stage. To improve the generalization performance of the model, we developed the model using the transfer learning technique. The constructed CNN was initialized by the pre-trained parameters on ImageNet-21k [24]. Supplementary Material 3 S3 shows the result of four tested backbones. The best-performing ResNet50 was adopted to develop the prediction model. Following the tricks proposed in big data transfer [25], we used group normalization and weight standardization instead of batch normalization in the ResNet50. The detailed structure of the network is presented in Supplementary Material 3 S4. During the training stage, we adopted the cross-entropy as the loss function, Adam optimizer with the initial learning rate of 0.003, and the learning rate multiplied by 0.1 every 100 epochs with the total epoch number: 500. Image augmentation was also used to alleviate overfitting. The images were randomly cropped, horizontally flipped and rotated in the range of [-20, 20] degrees.

### Development and explanation of the integrated prediction model

**Fig. 2:**
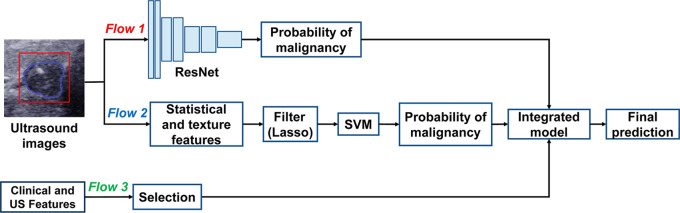
The flow chart of the artificial intelligence integrated model

The integrated prediction model mainly includes three branches, the deep learning branch, the hand-crafted radiomics branch, and the clinical and US feature branch. The flowchart outlining the integrated prediction model can be seen in Fig. 2. The deep learning branch was used to obtain the score value predicted by the ResNet50 with frozen parameters. In the hand-crafted radiomics branch, we adopted the predicted malignancy probability of the hand-crafted radiomics model for further integration. The already filtered clinical and US features were then used to create the final prediction model along with the predicted malignancy probabilities from the hand-crafted radiomics model and the deep learning model. We also employed a multivariable logistic regression for the integrated prediction model, with 10-fold cross-validation in the training set. To assess the performance of the integrated model, the performance of the model was compared with that of the hand-crafted radiomics model, DL model, and junior and senior radiologists on the independent test set.

In addition, the visualized explanation methods named SHapley Additive exPlanations (SHAP) plot and Gradient-weighted Class Activation Mapping (Grad-CAM) were applied to improve the clinical explanation of our model. We used Grad-CAM to extract the areas of interest and generate saliency maps for the DL model, while the SHAP plot was used to calculate the contribution value of each variable to the integrated model. These visualization methods aim to improve the clinical understanding and explanation of our model’s predictions.

### Statistical analysis

Statistical analysis was performed using IBM SPSS Statistics for Windows, Version 20.0 (IBM Corp.).

Categorical variables are presented as numbers and percentages and analyzed using the chi-squared or Fisher’s exact test. The Mann-Whitney U test analyzed continuous variables, and Kappa statistics analyzed the inter-observer agreement. The performance of predictive models was evaluated by the receiver operating characteristic (ROC) curve analysis and the area under curve (AUC). DeLong’s test compared the combined prediction model and other methods in predicting CLNM. Other performance measures, including accuracy, sensitivity, specificity, positive predictive value (PPV), and negative predictive value (NPV), were also assessed. The two-sided statistical significance was set at 0.05.

## Results

### Patient demographics and feature selection

Patient characteristics and US features of the thyroid nodules in the training and test cohorts were shown in Table 1. The training cohort included 460 patients (136 males, 324 females) with a mean age of 40.70 ± 11.16 years (range, 11–73 years). The independent test cohort included 153 patients (45 males and 108 females) with a mean age of 42.59 ± 11.33 years (range, 13–69 years). These two datasets were comparable as there were no significant differences. The inter-observer consistency was satisfactory, with Kappa coefficients between 0.82 and 0.92 (Supplementary Material 3 S5).

**Table 1:** Demographic comparison between training and independent test cohorts

Characteristics and US features	Training cohort (*n* = 460)	Independent test cohort (*n* = 153)	p value
Size (mean ± SD)	1.24 ± 0.92	1.16 ± 0.69	0.433
**Sex**			0.971
Male	136(75.1%)	45(24.9%)	
Female	324(75.0%)	108(25.0%)	
**Age**			0.484
≤ 55	415(75.5%)	135(24.5%)	
≤55	45(71.4%)	18(28.6%)	
**Tumour location**			0.711
Right lobe	228(73.8%)	81(26.2%)	
Left lobe	213(76.6%)	65(23.4%)	
Isthmus	19(73.1%)	7(26.9%)	
**Tumour position**			0.095
Upper	152(81.7%)	34(18.3%)	
Mid	164(71.9%)	64(28.1%)	
Lower	125(72.3%)	48(27.7%)	
Isthmus	19(73.1%)	7(26.9%)	
**Solid composition with hypoechoic echo**			0.110
Present	417(74.2%)	145(25.8%)	
Absent	43(84.3%)	8(15.7%)	
**Tumour multifocality**			0.769
Present	83(76.1%)	26(23.9%)	
Absent	377(74.8%)	127(25.2%)	
**Aspect ratio**			0.198
Present	201(726%)	76(27.4%)	
Absent	259(77.1%)	77(22.9%)	
**Microcalcification**			0.769
Present	360(75.3%)	118(24.7%)	
Absent	100(74.1%)	35(25.9%)	
**Tumour vascularity**			0.320
0–1	401(74.4%)	138(25.6%)	
2–3	59(79.7%)	15(20.3%)	
**Acoustic halo**			0.804
Present	42(73.7%)	15(26.3%)	
Absent	418(75.2%)	138(24.8%)	
**ETE**			0.947
Present	119(74.8%)	40(25.2%)	
Absent	341(75.1%)	113(24.9%)	
**Posterior acoustic attenuation**			0.282
Present	67(79.8%)	17(20.2%)	
Absent	393(74.3%)	136(25.7%)	
**CLNM in the pathology outcomes**			0.982
Present	228 (75.0%)	76 (25.0%)	
Absent	232 (75.1%)	77 (24.9%)	

*Abbreviations*: US, ultrasound; ETE, extrathyroidal extension; CLNM, central lymph node metastasis

To better understand the relationship between CLNM and clinical and US features, a multivariate analysis was performed in the training cohort. The results showed that age, sex, tumour size, multifocality, and ETE were independent risk factors for CLNM (Table 2).

**Table 2:** Independent risk factors after multiple logistic regression analysis

Ultrasound features	β	Odds ratio (95% CI)	p value
**Prediction of CLNM status**			
Size	0.533	1.704 (1.225–2.370)	0.002
Age	-1.301	0.272 (0.125–0.595)	0.001
Sex	-0.598	0.550 (0.353–0.857)	0.008
Tumour multifocality	0.738	2.092 (1.224–3.577)	0.007
ETE	1.418	4.130 (2.434–7.008)	< 0.001

*Abbreviations*: CLNM, central lymph node metastasis; US, ultrasound; CI: confidence interval; ETE, extrathyroidal extension

### Diagnostic performance of CLNM-predicting model

**Fig. 3:**
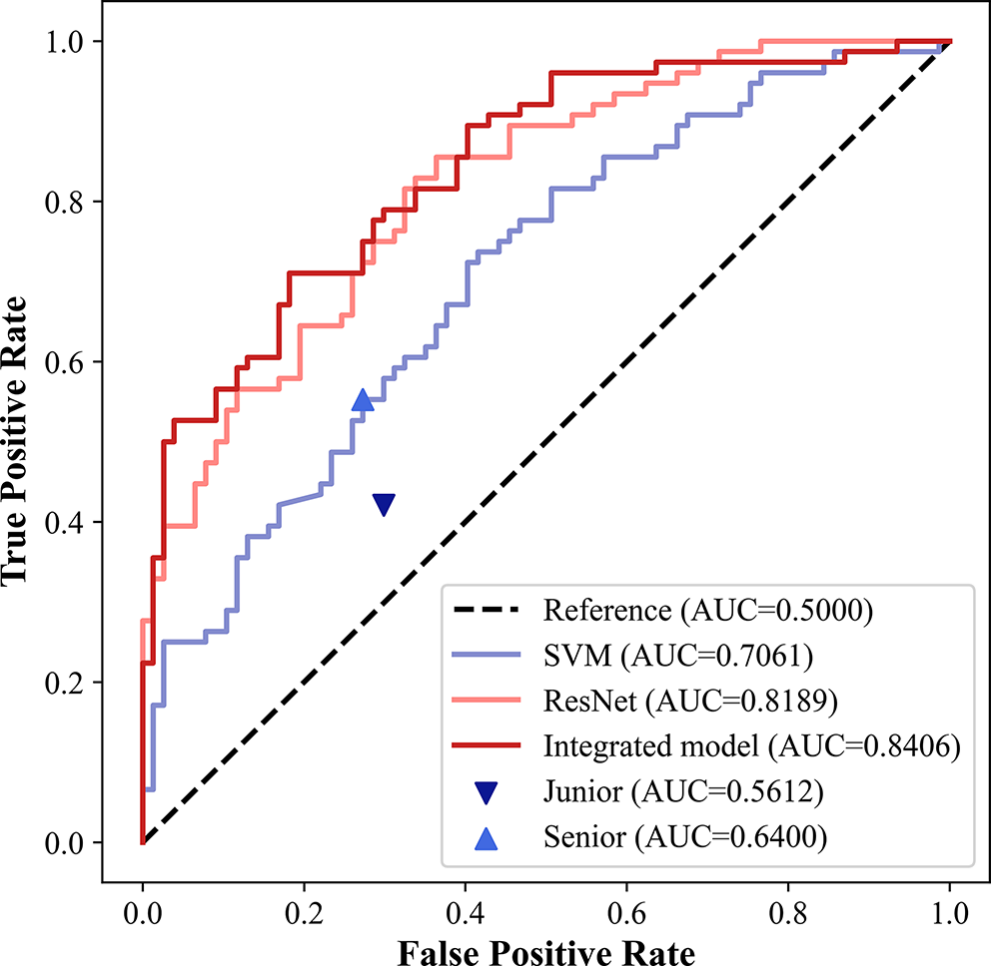
Diagnostic performance comparison among artificial intelligence models and radiologists in the independent testing cohort

We successfully built a hand-crafted radiomics model, a DL model, and an integrated model. In the testing set, our result showed that the DL model exhibited higher sensitivity (75.00% vs. 52.63%) but slightly lower specificity (71.43% vs. 74.03%) compared to the hand-crafted radiomics model (Table 3). By combining hand-crafted radiomics, DL and clinical features, the integrated model showed good predictive efficacy (the specificity and sensitivity were 81.82% and 72.37%, and the PPV and NPV were 79.71% and 75.00%). Meanwhile, the integrated model had most outstanding performance with the AUC of 0.841, which was superior to the hand-crafted radiomics model (0.841 vs. 0.706, *p* < 0.001) as well as the DL model (0.841 vs. 0.819, *p* = 0.26) (Fig. 3). These findings highlight the superior performance of the integrated model over the individual models.

**Table 3:** Performance comparison of different AI models in prediction of CLNM

Test cohort	AUC	95%CI	ACC (%)	SEN (%)	SPE (%)	PPV (%)	NPV (%)
ResNet	0.8189	[0.7542, 0.8835]	73.20	75.00	71.43	72.15	74.32
SVM	0.7061*	[0.6246, 0.7875]	63.40	52.63	74.03	66.67	61.29
Integrated Model	0.8406	[0.7792, 0.9020]	77.12	72.37	81.82	79.71	75.00

*Abbreviations*: CLNM, central lymph node metastasis; ACC, accuracy; SEN, sensitivity; SPE, specificity; PPV, positive predictive value; NPV, negative predictive value; AUC, area under the receiver operating curve; CI, confidence interval *Compared with integrated model, *p* < 0.05

### Performance comparison among integrated model and radiologists

The results indicated that the integrated model exhibited a significantly higher AUC compared to both junior and senior radiologists (0.841 vs. 0.561 and 0.640, *p* < 0.001). In comparison to the junior and senior radiologists, the integrated model demonstrated a decrease in the missed CLNM rate by 30.26% and 17.11% respectively. Additionally, the rate of unnecessary CLND decreased by 11.69% and 9.09%. A detailed comparison of the integrated model and radiologists were summarized in Table 4. These results indicated that integrated model could improve the efficiency of metastatic LNs detection and reduce the rate of unnecessary CLND.

**Table 4:** Performance comparison of radiologists and integrated model in prediction of CLNM

Test cohort	AUC	95%CI	Undetected CLNM (%)	Unnecessary CLND (%)	ACC (%)	SEN (%)	SPE (%)	PPV (%)	NPV (%)
Junior Radiologists	0.5612*	[0.4852, 0.6371]	57.89 (44/76)	29.87 (23/77)	56.21	42.11	70.13	58.18	55.10
Senior Radiologists	0.6400*	[0.5646, 0.7153]	44.74 (34/76)	27.27 (21/77)	64.05	55.26	72.73	66.67	62.22
Integrated Model	0.8406	[0.7792, 0.9020]	27.63 (21/76)	18.18 (14/77)	77.12	72.37	81.82	79.71	75.00

Abbreviations: CLNM, central lymph node metastasis; CLND, central lymph node dissection; ACC, accuracy; SEN, sensitivity; SPE, specificity; PPV, positive predictive value; NPV, negative predictive value; AUC, area under the receiver operating curve; CI, confidence interval *Compared with integrated model, *p* < 0.05

### Explanation of the integrated model

**Fig. 4:**
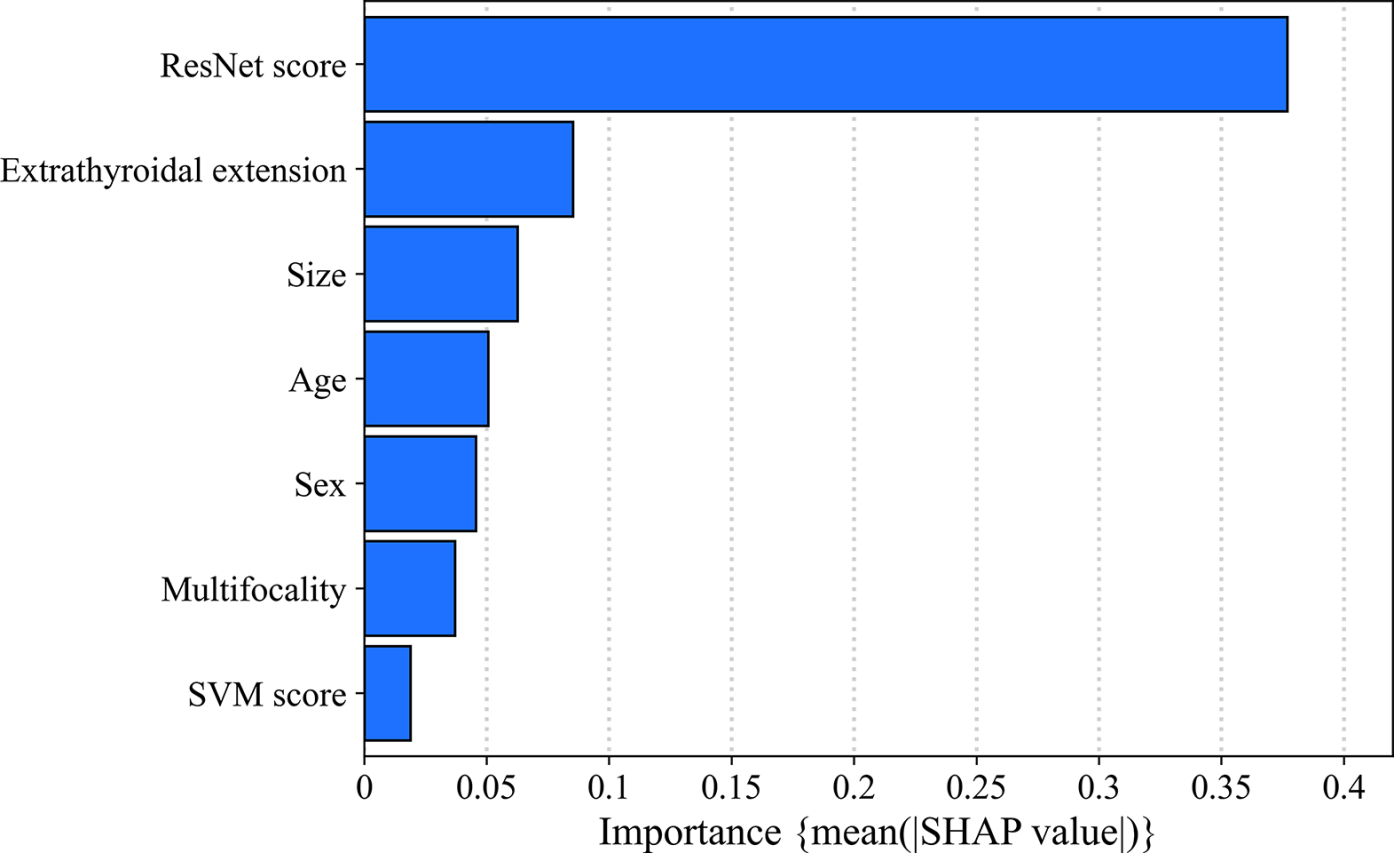
The SHAP plot reflected the contribution of each parameter to diagnose central lymph node metastasis in the integrated model

**Fig. 5:**
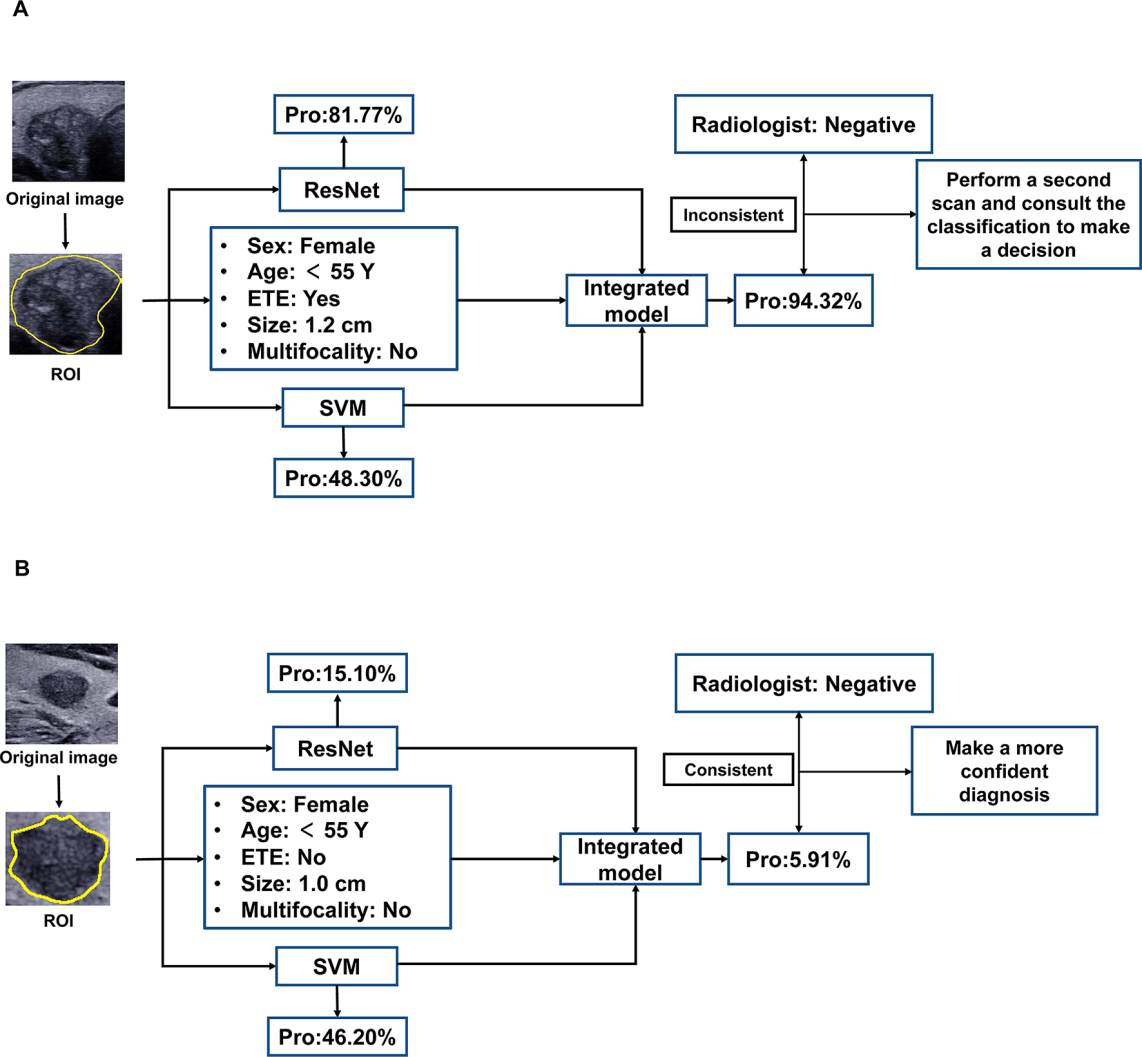
Two representative cases for the real output of the integrated model. (**a**). A 31-year-old female suffering from PTC with CLNM. The hand-crafted radiomics model outputs a probability of 48.30% for CLNM. The deep learning model outputs a probability of 81.77%, and the integrated model fuses the risk factors and gives a final probability of 94.32%. The result is inconsistent with the radiologist’s diagnosis, so the radiologist is recommended to conduct a second scan and then consult the classification provided by the integrated model. (**b**) A 28-year-old female suffering from PTC without CLNM. The hand-crafted radiomics model represented a probability of 46.20% for CLNM. The deep learning model and the integrated model output probabilities of 15.10% and 5.91%, respectively. The result is consistent with the diagnosis of the radiologist. CLNM, central lymph node metastasis; ETE, extrathyroidal extension; PTC, papillary thyroid carcinoma

**Fig. 6:**
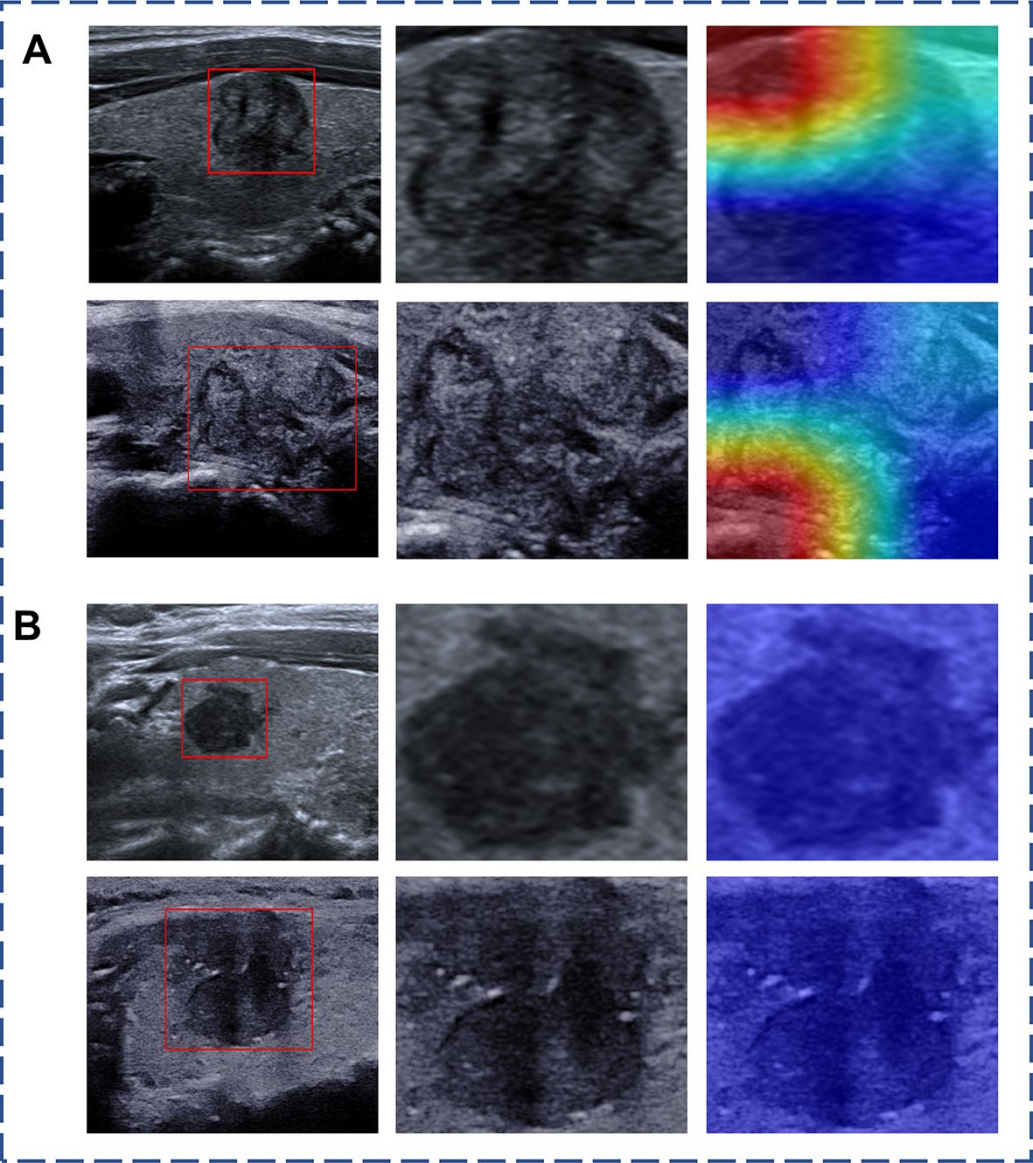
Representative examples of the saliency maps. (**A**) Saliency maps of one CLNM case evaluated by integrated model. The red colour highlighted the activation region associated with the thyroid capsule, consistent with the radiologists’ concentration on areas significantly associated with CLNM. (**B**) Saliency maps of a case without CLNM evaluated by integrated model. CLNM, central lymph node metastasis

To better compensate for the problem of “cognitive opacity” of AI models, we utilized SHAP plots to illustrate the contribution of each key parameter in the integrated model. The result showed that the DL model contributed the most to CLNM prediction, followed by ETE, tumour size, age, gender, and multifocality. The hand-crafted radiomics model played a relatively minor role within the integrated model (Fig. 4). In Fig. 5, two representative examples were presented to demonstrate how each key parameter contributed to the personalized decision-making process in the integrated model. Furthermore, we employed Grad-CAM to identify the areas of interest for the DL model. Figure 6 showcased several representative cases, indicating that the areas of interest were predominantly located around the thyroid capsule, consistent with the radiologists focusing on areas significantly associated with CLNM.

## Discussion

In this study, we developed an integrated model for predicting CLNM that incorporated deep learning, hand-crafted radiomics, and clinical and US features. Our integrated model outperformed models based solely on hand-crafted radiomics or DL features, as well as junior and senior radiologists. The integrated model decreased the rate of missed CLNM and unnecessary CLND, thus improving preoperative CLND decision-making. Furthermore, the integrated model’s visual explanation aligned with radiologists’ typical judgments, which contributed to the acceptance of AI-assisted US diagnosis.

Currently, one of the primary objectives of US in patients with PTC is to provide preoperative guidance for CLND by detecting the presence of CLNM. However, the presence of air interference in the trachea and esophagus, along with the small size of LNs, leads to unsatisfactory diagnostic accuracy [26]. Encouragingly, hand-crafted radiomics and DL methods can effectively reveal information that is imperceptible to the human eye, thereby enhancing diagnostic capabilities. Previous studies focusing solely on either hand-crafted radiomics or DL methods in diagnosing CLNM have yielded favourable results [27, 28]. However, our findings indicated limitations in the diagnostic efficacy of standalone DL and hand-crafted radiomics models. The DL model exhibited higher sensitivity, while the hand-crafted radiomics model showed higher specificity, indicating a distinction between traditional image features extracted by hand-crafted radiomics and the high-dimensional features extracted by DL. These observations motivated us to develop an integrated model that combines both types of features, resulting in superior performance compared to models based solely on hand-crafted radiomics or DL features. Furthermore, when compared with junior and senior radiologists, the integrated model significantly reduced the missed rate of CLNM by 30.26% and 17.11%, respectively, and decreased the rate of unnecessary CLND by 11.69% and 9.09%. Our findings indicate that the utilization of this model in clinical practice can be beneficial for PTC patients. Radiologists also can benefit from the integrated model, as it can serve as a valuable second opinion during the diagnosis of CLNM, assisting them in making more precise judgments and boosting their diagnostic confidence.

Consistent with the results of previous studies [29], we conducted a screening of clinical and US factors associated with CLNM during routine diagnostic work. These factors were then integrated into our AI model, resulting in improved efficacy. Upon further analysis using the SHAP plot, the integrated model demonstrated that the clinical and US factors provided valuable additional information. Among these crucial factors, ETE had the highest contribution, indicating that tumour cells could breach the thyroid capsule and enter the lymphatic system, leading to the development of metastatic LNs [30]. Additionally, tumour size, gender, age, and multifocality were also found to be associated with CLNM [31]. Interestingly, our findings revealed that the integrated model focused primarily on the thyroid capsule, which aligns with the areas of emphasis for radiologists when assessing CLNM. These results suggest that the clinical and US factors incorporated into the integrated model, as well as the regions of the model’s interest, are generally consistent with radiologists’ judgments, thereby providing the model with some clinical explainability. Overall, the visual explanation provided by the integrated model not only aligns with radiologists’ usual judgments but also the integrated model demonstrates higher diagnostic efficacy compared to radiologists. This enhances the clinical acceptance of AI-assisted US diagnosis among radiologists.

In contrast to the integrated models derived from CT or MRI images, where hand-crafted radiomics features played a prominent role [32, 33], our findings indicated that the contribution of hand-crafted radiomics features to our integrated model was relatively modest. This discrepancy may arise from the fact that some of the features extracted from US images through hand-crafted radiomics, such as shape, grayscale, and texture, can also be obtained through DL methods. Additionally, during US imaging, noise can be generated due to variations in signal intensity, which can degrade image quality and affect the extraction of certain hand-crafted radiomics features. Consequently, these circumstances may account for the relatively limited contribution of hand-crafted radiomics to the model.

Several limitations should be acknowledged in this study. Firstly, due to the interference of anatomical structures and the small size of the central LNs, US images of central LNs were not included in the analysis. Secondly, although the incorporation of clinical and US features enhances the acceptance of AI-assisted US diagnosis by radiologists, the interpretability of features learned by the DL and radiomics model remains limited. Future advancements in the field of interpretable AI will inspire further exploration. Finally, the results obtained may be influenced by the limited amount of data utilized. Further investigation of the value of integrated models in prospective studies with larger sample sizes is warranted.

In conclusion, the integrated model demonstrated superior performance compared to models relying solely on hand-crafted radiomics or DL features, exceeding the diagnostic capabilities of both junior and senior radiologists. The application of integrated models can significantly reduce missed CLNMs and unnecessary CLNDs along with increasing radiologists’ acceptance of AI-assisted US diagnoses.

### Electronic supplementary material

Below is the link to the electronic supplementary material.

**Supplementary Material 1:** CheckList for Artificial Intelligence in Medical Imaging (CLAIM)

**Supplementary Material 2:** CheckList for EvaluAtion of Radiomics research (CLEAR)

**Supplementary Material 3:** S1. Definition of extracted radiomic features. S2. Name of the extracted radiomics feature. S3. Performance comparison of the deep learning algorithms in the training and test datasets. S4. Structure of the ResNet50 used in the paper. S5. Intra-operator ultrasound feature measurement consistency

## Data Availability

The codes used during the current study can be accessed at https://github.com/yytangxiaoyuan/metastasis_predict_model/tree/main. Other analyzed datasets from the current study are not publicly available because the data contain information that may compromise patients, but are available from the corresponding author upon reasonable request.

## Abbreviations

AI: Artificial intelligence
AUC: Area under curve
CLND: Central lymph node dissection
CLNM: Central lymph node metastases
DL: Deep learning
Grad-CAM: Gradient-weighted Class Activation Mapping
LN: Lymph node
PTC: Papillary thyroid carcinoma
ROI: Region of interest
SHAP: SHapley Additive exPlanation
SVM: Support vector machine
