# Supplementary material for: An integrated model incorporating deep learning, hand-crafted radiomics and clinical and US features to diagnose central lymph node metastasis in patients with papillary thyroid cancer
Source: BMC Cancer. 2024 Jan 12;24:69. doi: 10.1186/s12885-024-11838-1 (PMC10787418; doi:10.1186/s12885-024-11838-1)
Supplement: Supplementary file 1 — Supplementary Material 1: CheckList for Artificial Intelligence in Medical Imaging (CLAIM) [file 12885_2024_11838_MOESM1_ESM.pdf]

CLAIM: Checklist for Artificial Intelligence in Medical Imaging

Section / Topic	No.	Item	
**TITLE / ABSTRACT**			
	**1**	Identification as a study of AI methodology, specifying the category of technology used (e.g., deep learning)	√
	**2**	Structured summary of study design, methods, results, and conclusions	√
**INTRODUCTION**			
	**3**	Scientific and clinical background, including the intended use and clinical role of the AI approach	√
	**4**	Study objectives and hypotheses	√
**METHODS**			
Study Design	**5**	Prospective or retrospective study	√
	**6**	Study goal, such as model creation, exploratory study, feasibility study, non-inferiority trial	√
Data	**7**	Data sources	√
	**8**	Eligibility criteria: how, where, and when potentially eligible participants or studies were identified (e.g., symptoms, results from previous tests, inclusion in registry, patient-care setting, location, dates)	√
	**9**	Data pre-processing steps	√
	**10**	Selection of data subsets, if applicable	√
	**11**	Definitions of data elements, with references to Common Data Elements	√
	**12**	De-identification methods	**N/A**
	**13**	How missing data were handled	**N/A**
Ground Truth	**14**	Definition of ground truth reference standard, in sufficient detail to allow replication	√
	**15**	Rationale for choosing the reference standard (if alternatives exist)	√
	**16**	Source of ground-truth annotations; qualifications and preparation of annotators	√
	**17**	Annotation tools	√
	**18**	Measurement of inter- and intrarater variability; methods to mitigate variability and/or resolve discrepancies	√
Data Partitions	**19**	Intended sample size and how it was determined	√
	**20**	How data were assigned to partitions; specify proportions	√
	**21**	Level at which partitions are disjoint (e.g., image, study, patient, institution)	**N/A**

Model	**22**	Detailed description of model, including inputs, outputs, all intermediate layers and connections	√
	**23**	Software libraries, frameworks, and packages	√
	**24**	Initialization of model parameters (e.g., randomization, transfer learning)	√
Training	**25**	Details of training approach, including data augmentation, hyperparameters, number of models trained	√
	**26**	Method of selecting the final model	√
	**27**	Ensembling techniques, if applicable	√
Evaluation	**28**	Metrics of model performance	√
	**29**	Statistical measures of significance and uncertainty (e.g., confidence intervals)	√
	**30**	Robustness or sensitivity analysis	√
	**31**	Methods for explainability or interpretability (e.g., saliency maps), and how they were validated	√
	**32**	Validation or testing on external data	√
**RESULTS**			
Data	**33**	Flow of participants or cases, using a diagram to indicate inclusion and exclusion	√
	**34**	Demographic and clinical characteristics of cases in each partition	√
Model performance	**35**	Performance metrics for optimal model(s) on all data partitions	√
	**36**	Estimates of diagnostic accuracy and their precision (such as 95% confidence intervals)	√
	**37**	Failure analysis of incorrectly classified cases	√
**DISCUSSION**			
	**38**	Study limitations, including potential bias, statistical uncertainty, and generalizability	√
	**39**	Implications for practice, including the intended use and/or clinical role	√
**OTHER INFORMATION**			
	**40**	Registration number and name of registry	√
	**41**	Where the full study protocol can be accessed	√
	**42**	Sources of funding and other support; role of funders	√

Mongan J, Moy L, Kahn CE Jr. Checklist for Artificial Intelligence in Medical Imaging (CLAIM): a guide for authors and reviewers. Radiol Artif Intell 2020; 2(2):e200029. https://doi.org/10.1148/ryai.2020200029

RSNA®
