# Supplementary material for: An integrated model incorporating deep learning, hand-crafted radiomics and clinical and US features to diagnose central lymph node metastasis in patients with papillary thyroid cancer
Source: BMC Cancer. 2024 Jan 12;24:69. doi: 10.1186/s12885-024-11838-1 (PMC10787418; doi:10.1186/s12885-024-11838-1)
Supplement: Supplementary file 2 — Supplementary Material 2: CheckList for EvaluAtion of Radiomics research (CLEAR) [file 12885_2024_11838_MOESM2_ESM.pdf]

CLEAR Checklist v1.0

Note: Use the checklist in conjunction with the main text for clarification of all items.
Yes, details provided; No, details not provided; n/e, not essential; n/a, not applicable; Page, page number

Section	No.	Item	Yes	No	n/a	Page
**Title**						
	1	Relevant title, specifying the radiomic methodology	☑	☐	☐	1
**Abstract**						
	2	Structured summary with relevant information	☑	☐	☐	2
**Keywords**						
	3	Relevant keywords for radiomics	☑	☐	☐	3
**Introduction**						
	4	Scientific or clinical background	☑	☐	☐	4
	5	Rationale for using a radiomic approach	☑	☐	☐	5
	6	Study objective(s)	☑	☐	☐	5
**Method**						
Study Design	7	Adherence to guidelines or checklists (e.g., CLEAR checklist)	☑	☐	☐	6
	8	Ethical details (e.g., approval, consent, data protection)	☑	☐	☐	6
	9	Sample size calculation	☑	☐	☐	6
	10	Study nature (e.g., retrospective, prospective)	☑	☐	☐	6
	11	Eligibility criteria	☑	☐	☐	6-7
	12	Flowchart for technical pipeline	☑	☐	☐	10
Data	13	Data source (e.g., private, public)	☑	☐	☐	7
	14	Data overlap	☐	☐	☑	
	15	Data split methodology	☑	☐	☐	7
	16	Imaging protocol (i.e., image acquisition and processing)	☑	☐	☐	7-8
	17	Definition of non-radiomic predictor variables	☑	☐	☐	7-8
	18	Definition of the reference standard (i.e., outcome variable)	☑	☐	☐	7-8
Segmentation	19	Segmentation strategy	☑	☐	☐	9
	20	Details of operators performing segmentation	☑	☐	☐	9
Pre-processing	21	Image pre-processing details	☑	☐	☐	9-10
	22	Resampling method and its parameters	☑	☐	☐	9-10
	23	Discretization method and its parameters	☑	☐	☐	9

Section	No.	Item	Yes	No	n/a	Page
	24	Image types (e.g., original, filtered, transformed)	☑	☐	☐	9
Feature extraction	25	Feature extraction method	☑	☐	☐	9-10
	26	Feature classes	☑	☐	☐	9-10
	27	Number of features	☑	☐	☐	9-10
	28	Default configuration statement for remaining parameters	☑	☐	☐	9-10
Data preparation	29	Handling of missing data	☐	☐	☑	
	30	Details of class imbalance	☐	☐	☑	
	31	Details of segmentation reliability analysis	☑	☐	☐	9
	32	Feature scaling details (e.g., normalization, standardization)	☑	☐	☐	9-10
	33	Dimension reduction details	☑	☐	☐	9-10
Modeling	34	Algorithm details	☑	☐	☐	9-10
	35	Training and tuning details	☑	☐	☐	9-10
	36	Handling of confounders	☐	☐	☑	
	37	Model selection strategy	☑	☐	☐	9-10
Evaluation	38	Testing technique (e.g., internal, external)	☑	☐	☐	11
	39	Performance metrics and rationale for choosing	☑	☐	☐	12
	40	Uncertainty evaluation and measures (e.g., confidence intervals)	☑	☐	☐	9-10
	41	Statistical performance comparison (e.g., DeLong's test)	☑	☐	☐	12
	42	Comparison with non-radiomic and combined methods	☑	☐	☐	11
	43	Interpretability and explainability methods	☑	☐	☐	11
**Results**						
	44	Baseline demographic and clinical characteristics	☑	☐	☐	12
	45	Flowchart for eligibility criteria	☑	☐	☐	7
	46	Feature statistics (e.g., reproducibility, feature selection)	☑	☐	☐	10
	47	Model performance evaluation	☑	☐	☐	13
	48	Comparison with non-radiomic and combined approaches	☑	☐	☐	14
**Discussion**						
	49	Overview of important findings	☑	☐	☐	17
	50	Previous works with differences from the current study	☑	☐	☐	19
	51	Practical implications	☑	☐	☐	18
	52	Strengths and limitations (e.g., bias and generalizability issues)	☑	☐	☐	20

Section	No.	Item	Yes	No	n/a	Page
**Open Science**						
Data availability	53	Sharing images along with segmentation data [n/e]	☐	☐	☑	
	54	Sharing radiomic feature data	☑	☐	☐	21
Code availability	55	Sharing pre-processing scripts or settings	☑	☐	☐	21
	56	Sharing source code for modeling	☑	☐	☐	21
Model availability	57	Sharing final model files	☑	☐	☐	21
	58	Sharing a ready-to-use system [n/e]	☐	☐	☑	

Kocak B, Baessler B, Bakas S, Cuocolo R, Fedorov A, Maier-Hein L, Mercaldo N, Müller H, Orlhac F, Pinto Dos Santos D, Stanzione A, Ugga L, Zwanenburg A. CheckList for EvaluAtion of Radiomics research (CLEAR): a step-by-step reporting guideline for authors and reviewers endorsed by ESR and EuSoMII. Insights Imaging. 2023 May 4;14(1):75. doi: 10.1186/s13244-023-01415-8
