# Supplementary material for: An integrated model incorporating deep learning, hand-crafted radiomics and clinical and US features to diagnose central lymph node metastasis in patients with papillary thyroid cancer
Source: BMC Cancer. 2024 Jan 12;24:69. doi: 10.1186/s12885-024-11838-1 (PMC10787418; doi:10.1186/s12885-024-11838-1)
Supplement: Supplementary file 3 — Supplementary Material 3: S1. Definition of extracted radiomic features. S2. Name of the extracted radiomics feature. S3. Performance comparison of the deep learning algorithms in the training and test datasets. S4. Structure of the ResNet50 used in the paper. S5. Intra-operator ultrasound feature measurement consistency [file 12885_2024_11838_MOESM3_ESM.docx]

**Supplemental S1** Definition of extracted radiomic features.

We extracted 783 radiomic features from each delineated ROI using the open-source python package pyradiomics (version 3.1.0). The images were resampled to 1×1 mm to standardize the pixel spacing. The number of discretized bin was set to 25. These features can be divided into five groups: first-order statistics, texture features, shape features, wavelet decompositions, and Laplacian of Gaussian features. The definition can also be found on https://pyradiomics.readthedocs.io/en/latest/.

*Group 1: First-order statistics*

First-order statistics describe the distribution of voxel intensities within the image region defined by the mask through commonly used and basic metrics.

Let:

- **X** be a set of ***N****_p_* voxels included in the ROI.
- **P**(*i*) be the first order histogram with **N**_g_ discrete intensity levels, where **N**_g_ is the number of non-zero bins, equally spaced from 0 with a width defined in the binWidth parameter.
- *p*(*i*) be the normalized first order histogram and equal to **P**(*i*) / ***N****_p_*.

1. Energy:

$$energy=\sum_{i=1}^{N_{p}} \left(X\left(i\right)+c\right)^{2}$$

2. Total energy:

$$total energy=V_{voxel}\sum_{i=1}^{N_{p}} \left(X\left(i\right)+c\right)^{2}$$

3. Entropy:

$$entropy=-\sum_{i=1}^{N_{g}} p\left(i\right)\log_{2}\left(p\left(i\right)+\varepsilon\right)$$

4. Minimum:

*minimum* = min(**X**)

5. 10th percentile: The 10^th^ percentile of **X**.

6. 90th percentile: The 90^th^ percentile of **X**.

7. Maximum:

*maximum* = max(**X**)

8. Mean:

$$mean=\frac{1}{N_{p}}\sum_{i=1}^{N_{p}} X\left(i\right)$$

9. Median: The median gray level intensity within the ROI.

10. Interquartile range:

*interquartile range* = **P**_75_ – **P**_25_

11. Range:

*range* = max(**X**) – min(**X**)

12. Mean absolute deviation:

$$MAD=\frac{1}{N_{p}}\sum_{i=1}^{N_{p}} \left|X\left(i\right)-\bar{X}\right|$$

13. Robust mean absolute deviation:

$$rMAD=\frac{1}{N_{10-90}}\sum_{i=1}^{N_{10-90}} \left|X_{10-90}\left(i\right)-\bar{X}_{10-90}\right|$$

14. Root mean squared:

$$RMS=\sqrt{\frac{1}{N_{p}}\sum_{i=1}^{N_{p}} \left(X\left(i\right)+c\right)^{2}}$$

15. Skewness:

$$skewness=\frac{\mu_{3}}{\sigma^{3}}=\frac{\frac{1}{N_{p}}\sum_{i=1}^{N_{p}} \left(X\left(i\right)-\bar{X}\right)^{3}}{\left(\frac{1}{N_{p}}\sum_{i=1}^{N_{p}} \left(X\left(i\right)-\bar{X}\right)^{2}\right)^{3}}$$

16. Kurtosis:

$$skewness=\frac{\mu_{4}}{\sigma^{4}}=\frac{\frac{1}{N_{p}}\sum_{i=1}^{N_{p}} \left(X\left(i\right)-\bar{X}\right)^{4}}{\left(\frac{1}{N_{p}}\sum_{i=1}^{N_{p}} \left(X\left(i\right)-\bar{X}\right)^{2}\right)^{2}}$$

17. Variance:

$$variance=\frac{1}{N_{p}}\sum_{i=1}^{N_{p}} \left(X\left(i\right)-\bar{X}\right)^{2}$$

18. Uniformity

$$uniformity=\sum_{i=1}^{N_{g}} p\left(i\right)^{2}$$

*Group 2: Texture features*

Textural features describe the spatial distribution of voxel intensities and are calculated from four matrices: the gray level co-occurrence matrix (GLCM), the gray-level run-length matrix (GLRLM), the gray-level size zone matrix (GLSZM), and the gray-level dependence matrix (GLDM). The intensity values within the ROI were discretized using a bin width of 25 Hounsfield units.

*Gray Level Co-occurrence Matrix (GLCM) features*

A Gray Level Co-occurrence Matrix (GLCM) of size **N**_g_ × **N**_g_ describes the second-order joint probability function of an image region constrained by the mask and is defined as **P**(*i*, *j*|*δ*, *θ*). The (*i*, *j*)^th^ element of this matrix represents the number of times the combination of levels *i* and *j* occur in two pixels in the image, that are separated by a distance of *δ* pixels along angle *θ*. The distance *δ* from the center voxel is defined as the distance according to the infinity norm.

Let:

- $\varepsilon$ be an arbitrarily small positive number ($\approx$2.2$\times$10^-16^).
- **P**(*i*, *j*) be the co-occurrence matrix for an arbitrary *δ* and *θ*.
- *p*(*i*, *j*) be the normalized co-occurrence matrix and equal to **P**(*i*, *j*) / Σ**P**(*i*, *j*).
- **N**_g_ be the number of discrete intensity levels in the image.
- $p_{x}\left(i\right)=\sum_{i=1}^{N_{g}} p\left(i,j\right)$ be the marginal row probabilities.
- $p_{y}\left(j\right)=\sum_{i=1}^{N_{g}} p\left(i,j\right)$ be the marginal column probabilities.
- $\mu_{x}$ be the mean gray level intensity of $p_{x}$ and defined as $\mu_{x}=\sum_{i=1}^{N_{g}} p_{x}\left(i\right) i$.
- $\mu_{y}$ be the mean gray level intensity of $p_{y}$ and defined as $\mu_{y}=\sum_{j=1}^{N_{g}} p_{y}\left(j\right) j$.
- *σ_x_* be the standard deviation of *p_x_*.
- *σ_y_* be the standard deviation of *p_y_*.
- $p_{x+y}\left(k\right)=\sum_{i=1}^{N_{g}} \sum_{j=1}^{N_{g}} p\left(i,j\right)$, where *i* + *j* = *k*, and *k* = 2, 3, …, 2*N_g_*.
- $p_{x-y}\left(k\right)=\sum_{i=1}^{N_{g}} \sum_{j=1}^{N_{g}} p\left(i,j\right)$, where |*i* – *j*| = *k*, and *k* = 0, 1, …, *N_g_* – 1.
- $HX=-\sum_{i=1}^{N_{g}} p_{x}\left(i\right)\log_{2}\left(p_{x}\left(i\right)+\varepsilon\right)$ be the entropy of *p_x_*.
- $HY=-\sum_{j=1}^{N_{g}} p_{y}\left(j\right)\log_{2}\left(p_{y}\left(j\right)+\varepsilon\right)$ be the entropy of *p_y_*.
- $HXY=-\sum_{i=1}^{N_{g}} \sum_{j=1}^{N_{g}} p\left(i,j\right)\log_{2}\left(p\left(i,j\right)+\varepsilon\right)$ be the entropy of *p*(*i, j*).
- $HXY1=-\sum_{i=1}^{N_{g}} \sum_{j=1}^{N_{g}} p\left(i,j\right)\log_{2}\left(p_{x}\left(i\right)p_{y}\left(j\right)+\varepsilon\right)$.
- $HXY2=-\sum_{i=1}^{N_{g}} \sum_{j=1}^{N_{g}} p_{x}\left(i\right)p_{y}\left(j\right)\log_{2}\left(p_{x}\left(i\right)p_{y}\left(j\right)+\varepsilon\right)$.

1. Autocorrelation:

$$autocorrelation=\sum_{i=1}^{N_{g}} \sum_{j=1}^{N_{g}} p\left(i,j\right)ij$$

2. Joint average:

$$joint average=\mu_{x}=\sum_{i=1}^{N_{g}} \sum_{j=1}^{N_{g}} p\left(i,j\right)i$$

3. Cluster prominence:

$$cluster prominence=\sum_{i=1}^{N_{g}} \sum_{j=1}^{N_{g}} \left(i+j-\mu_{x}-\mu_{y}\right)^{4}p\left(i,j\right)$$

4. Cluster shade:

$$cluster shade=\sum_{i=1}^{N_{g}} \sum_{j=1}^{N_{g}} \left(i+j-\mu_{x}-\mu_{y}\right)^{3}p\left(i,j\right)$$

5. Cluster tendency:

$$cluster tendency=\sum_{i=1}^{N_{g}} \sum_{j=1}^{N_{g}} \left(i+j-\mu_{x}-\mu_{y}\right)^{2}p\left(i,j\right)$$

6. Contrast:

$$contrast=\sum_{i=1}^{N_{g}} \sum_{j=1}^{N_{g}} \left(i-j\right)^{2}p\left(i,j\right)$$

7. Correlation:

$$correlation=\frac{\sum_{i=1}^{N_{g}} \sum_{j=1}^{N_{g}} p\left(i,j\right)ij-\mu_{x}\mu_{y}}{\sigma_{x}\left(i\right)\sigma_{y}\left(j\right)}$$

8. Difference average:

$$difference average=\sum_{k=0}^{N_{g}-1} kp_{x-y}\left(k\right)$$

9. Difference entropy:

$$difference entropy=\sum_{k=0}^{N_{g}-1} p_{x-y}\left(k\right)\log_{2}\left(p_{x-y}\left(k\right)+\varepsilon\right)$$

10. Difference variance:

$$difference variance=\sum_{k=0}^{N_{g}-1} \left(k-DA\right)^{2}p_{x-y}\left(k\right)$$

11. Joint energy:

$$joint energy=\sum_{i=1}^{N_{g}} \sum_{j=1}^{N_{g}} \left(p\left(i,j\right)\right)^{2}$$

12. Joint entropy:

$$joint entropy=-\sum_{i=1}^{N_{g}} \sum_{j=1}^{N_{g}} p\left(i,j\right)\log_{2}\left(p\left(i,j\right)+\varepsilon\right)$$

13. Informational measure of correlation 1:

$$IMC 1=\frac{HXY-HXY1}{\max\left\{HX,HY\right\}}$$

14. Informational measure of correlation 2:

$$IMC 2=\sqrt{1-e^{-2\left(HXY2-HXY\right)}}$$

15. Inverse difference moment:

$$IDM=\sum_{k=0}^{N_{g}-1} \frac{p_{x-y}\left(k\right)}{1+k^{2}}$$

16. Inverse difference moment normalized:

$$IDMN=\sum_{k=0}^{N_{g}-1} \frac{p_{x-y}\left(k\right)}{1+\left(\frac{k^{2}}{N_{g}^{2}}\right)}$$

17. Inverse difference:

$$ID=\sum_{k=0}^{N_{g}-1} \frac{p_{x-y}\left(k\right)}{1+k}$$

18. Inverse difference normalized:

$$IDN=\sum_{k=0}^{N_{g}-1} \frac{p_{x-y}\left(k\right)}{1+\left(\frac{k}{N_{g}}\right)}$$

19. Inverse variance:

$$inverse variance=\sum_{k=1}^{N_{g}-1} \frac{p_{x-y}\left(k\right)}{k^{2}}$$

20. Maximum probability:

$$maximum probability=\max\left(p\left(i,j\right)\right)$$

21. Sum entropy:

$$sum entropy=\sum_{k=2}^{2N_{g}} p_{x+y}\left(k\right)\log_{2}\left(p_{x+y}\left(k\right)+\varepsilon\right)$$

22. Sum of squares:

$$sum squares=\sum_{i=1}^{N_{g}} \sum_{j=1}^{N_{g}} \left(i-\mu_{x}\right)^{2}p\left(i,j\right)$$

*Gray-Level Run-Length Matrix (GLRLM) features*

A Gray Level Run Length Matrix (GLRLM) quantifies gray level runs, which are defined as the length in number of pixels, of consecutive pixels that have the same gray level value. In a gray level run length matrix **P**(*i*, *j*|*θ*), the (*i*, *j*)^th^ element describes the number of runs with gray level *i* and length *j* occur in the image (ROI) along angle *θ*.

Let:

- **N**_g_ be the number of discrete intensity levels in the image.
- **N**_r_ be the number of discrete run lengths in the image.
- **N**_p_ be the number of voxels in the image.
- **N**_r_ (*θ*) be the number of runs in the image along angle *θ*, which is equal to $\sum_{i=1}^{N_{g}} \sum_{j=1}^{N_{r}} p\left(i,j|\theta\right)$ and $1\leq N_{r}\left(\theta\right)\leq N_{p}$.
- **P***(i, j|θ)* be the run length matrix for an arbitrary direction *θ*.
- *p(i, j|θ)* be the normalized run length matrix, defined as $p\left(i,j|\theta\right)=\frac{P\left(i,j|\theta\right)}{N_{r}\left(\theta\right)}$.

1. Short run emphasis:

$$SRE=\frac{\sum_{i=1}^{N_{g}} \sum_{j=1}^{N_{r}} \frac{P\left(i,j|\theta\right)}{j^{2}}}{N_{r}\left(\theta\right)}$$

2. Long run emphasis:

$$LRE=\frac{\sum_{i=1}^{N_{g}} \sum_{j=1}^{N_{r}} P\left(i,j|\theta\right)j^{2}}{N_{r}\left(\theta\right)}$$

3. Gray level non-uniformity:

$$GLN=\frac{\sum_{i=1}^{N_{g}} \left(\sum_{j=1}^{N_{r}} P\left(i,j|\theta\right)\right)^{2}}{N_{r}\left(\theta\right)}$$

4. Gray level non-uniformity normalized:

$$GLNN=\frac{\sum_{i=1}^{N_{g}} \left(\sum_{j=1}^{N_{r}} P\left(i,j|\theta\right)\right)^{2}}{N_{r}\left(\theta\right)^{2}}$$

5. Run length non-uniformity:

$$RLN=\frac{\sum_{j=1}^{N_{r}} \left(\sum_{i=1}^{N_{g}} P\left(i,j|\theta\right)\right)^{2}}{N_{r}\left(\theta\right)}$$

6. Run length non-uniformity normalized:

$$RLNN=\frac{\sum_{j=1}^{N_{r}} \left(\sum_{i=1}^{N_{g}} P\left(i,j|\theta\right)\right)^{2}}{N_{r}\left(\theta\right)^{2}}$$

7. Run percentage:

$$RP=\frac{N_{r}\left(\theta\right)}{N_{p}}$$

8. Gray level variance:

$$GLV=\sum_{i=1}^{N_{g}} \sum_{j=1}^{N_{r}} p\left(i,j|\theta\right)\left(i-\mu\right)^{2}$$

9. Run variance:

$$RV=\sum_{i=1}^{N_{g}} \sum_{j=1}^{N_{r}} p\left(i,j|\theta\right)\left(j-\mu\right)^{2}$$

10. Run entropy:

$$RE=-\sum_{i=1}^{N_{g}} \sum_{j=1}^{N_{r}} p\left(i,j|\theta\right)\log_{2}\left(p\left(i,j|\theta\right)+\varepsilon\right)$$

11. Low gray level run emphasis:

$$LGLRE=\frac{\sum_{i=1}^{N_{g}} \sum_{j=1}^{N_{r}} \frac{P\left(i,j|\theta\right)}{i^{2}}}{N_{r}\left(\theta\right)}$$

12. High gray level run emphasis:

$$HGLRE=\frac{\sum_{i=1}^{N_{g}} \sum_{j=1}^{N_{r}} P\left(i,j|\theta\right)i^{2}}{N_{r}\left(\theta\right)}$$

13. Short run low gray level emphasis:

$$SRLGLE=\frac{\sum_{i=1}^{N_{g}} \sum_{j=1}^{N_{r}} \frac{P\left(i,j|\theta\right)}{i^{2}j^{2}}}{N_{r}\left(\theta\right)}$$

14. Short run high gray level emphasis:

$$SRHGLE=\frac{\sum_{i=1}^{N_{g}} \sum_{j=1}^{N_{r}} \frac{P\left(i,j|\theta\right)i^{2}}{j^{2}}}{N_{r}\left(\theta\right)}$$

15. Long run low gray level emphasis:

$$LRLGLRE=\frac{\sum_{i=1}^{N_{g}} \sum_{j=1}^{N_{r}} \frac{P\left(i,j|\theta\right)j^{2}}{i^{2}}}{N_{r}\left(\theta\right)}$$

16. Long run high gray level emphasis:

$$LRHGLRE=\frac{\sum_{i=1}^{N_{g}} \sum_{j=1}^{N_{r}} P\left(i,j|\theta\right)i^{2}j^{2}}{N_{r}\left(\theta\right)}$$

*Gray-Level Size Zone Matrix (GLSZM) features*

A Gray Level Size Zone (GLSZM) quantifies gray level zones in an image. A gray level zone is defined as a the number of connected voxels that share the same gray level intensity. A voxel is considered connected if the distance is 1 according to the infinity norm (26-connected region in a 3D, 8-connected region in 2D). In a gray level size zone matrix **P**(*i*, *j*) the (*i*, *j*)^th^ element equals the number of zones with gray level *i* and size *j* appear in image. Contrary to GLCM and GLRLM, the GLSZM is rotation independent, with only one matrix calculated for all directions in the ROI.

Let:

- **N**_g_ be the number of discrete intensity levels in the image.
- **N**_s_ be the number of discrete zone sizes in the image.
- **N**_p_ be the number of voxels in the image.
- **N**_z_ be the number of zones in the ROI, which is equal to $\sum_{i=1}^{N_{g}} \sum_{j=1}^{N_{s}} P\left(i,j\right)$ and $1\leq N_{z}\leq N_{p}$.
- **P**(*i, j*) be the size zone matrix.
- *p*(*i, j*) be the normalized size zone matrix, defined as $p\left(i,j\right)=\frac{P\left(i,j\right)}{N_{z}}$.

1. Small area emphasis:

$$SAE=\frac{\sum_{i=1}^{N_{g}} \sum_{j=1}^{N_{s}} \frac{P\left(i,j\right)}{j^{2}}}{N_{z}}$$

2. Large area emphasis:

$$LAE=\frac{\sum_{i=1}^{N_{g}} \sum_{j=1}^{N_{s}} P\left(i,j\right)j^{2}}{N_{z}}$$

3. Gray level non-uniformity:

$$GLN=\frac{\sum_{i=1}^{N_{g}} \left(\sum_{j=1}^{N_{s}} P\left(i,j\right)\right)^{2}}{N_{z}}$$

4. Gray level non-uniformity normalized:

$$GLNN=\frac{\sum_{i=1}^{N_{g}} \left(\sum_{j=1}^{N_{s}} P\left(i,j\right)\right)^{2}}{N_{z}^{2}}$$

5. Size zone non-uniformity:

$$SZN=\frac{\sum_{j=1}^{N_{s}} \left(\sum_{i=1}^{N_{g}} P\left(i,j\right)\right)^{2}}{N_{z}}$$

6. Size zone non-uniformity normalized:

$$SZNN=\frac{\sum_{j=1}^{N_{s}} \left(\sum_{i=1}^{N_{g}} P\left(i,j\right)\right)^{2}}{N_{z}^{2}}$$

7. Zone percentage:

$$ZP=\frac{N_{z}}{N_{p}}$$

8. Gray level variance:

$$GLV=\sum_{i=1}^{N_{g}} \sum_{j=1}^{N_{s}} p\left(i,j\right)\left(i-\mu\right)^{2}$$

9. Zone variance:

$$ZV=\sum_{i=1}^{N_{g}} \sum_{j=1}^{N_{s}} p\left(i,j\right)\left(j-\mu\right)^{2}$$

10. Zone entropy:

$$ZE=-\sum_{i=1}^{N_{g}} \sum_{j=1}^{N_{s}} p\left(i,j\right)\log_{2}\left(p\left(i,j\right)+\varepsilon\right)$$

11. Low gray level zone emphasis:

$$LGLZE=\frac{\sum_{i=1}^{N_{g}} \sum_{j=1}^{N_{s}} \frac{P\left(i,j\right)}{i^{2}}}{N_{z}}$$

12. High gray level zone emphasis:

$$HGLZE=\frac{\sum_{i=1}^{N_{g}} \sum_{j=1}^{N_{s}} P\left(i,j\right)i^{2}}{N_{z}}$$

13. Small area low gray level emphasis:

$$SALGLE=\frac{\sum_{i=1}^{N_{g}} \sum_{j=1}^{N_{s}} \frac{P\left(i,j\right)}{i^{2}j^{2}}}{N_{z}}$$

14. Small area high gray level emphasis:

$$SAHGLE=\frac{\sum_{i=1}^{N_{g}} \sum_{j=1}^{N_{s}} \frac{P\left(i,j\right)i^{2}}{j^{2}}}{N_{z}}$$

15. Large area low gray level emphasis:

$$LALGLE=\frac{\sum_{i=1}^{N_{g}} \sum_{j=1}^{N_{s}} \frac{P\left(i,j\right)j^{2}}{i^{2}}}{N_{z}}$$

16. Large area high gray level emphasis:

$$LAHGLE=\frac{\sum_{i=1}^{N_{g}} \sum_{j=1}^{N_{s}} P\left(i,j\right)i^{2}j^{2}}{N_{z}}$$

*Gray-Level Dependence Matrix (GLDM) feature:*

A Gray Level Dependence Matrix (GLDM) quantifies gray level dependencies in an image. A gray level dependency is defined as a the number of connected voxels within distance *δ* that are dependent on the center voxel. A neighbouring voxel with gray level *j* is considered dependent on center voxel with gray level *i* if |*i* − *j*| ≤ *α*. In a gray level dependence matrix **P**(*i*, *j*) the (*i*, *j*)^th^ element describes the number of times a voxel with gray level *i* with *j* dependent voxels in its neighbourhood appears in image.

Let:

- **N**_g_ be the number of discrete intensity levels in the image.
- **N**_d_ be the number of discrete dependency sizes in the image.
- **N**_z_ be the number of dependency zones in the image, which is equal to $\sum_{i=1}^{N_{g}} \sum_{j=1}^{N_{d}} P\left(i,j\right)$.
- **P**(*i, j*) be the dependence matrix.
- *p*(*i, j*) be the normalized dependence matrix, defined as $p\left(i,j\right)=\frac{P\left(i,j\right)}{N_{z}}$.

1. Small dependence emphasis:

$$SDE=\frac{\sum_{i=1}^{N_{g}} \sum_{j=1}^{N_{d}} \frac{P\left(i,j\right)}{i^{2}}}{N_{z}}$$

2. Large dependence emphasis:

$$LDE=\frac{\sum_{i=1}^{N_{g}} \sum_{j=1}^{N_{d}} P\left(i,j\right)j^{2}}{N_{z}}$$

3. Gray level non-uniformity:

$$GLN=\frac{\sum_{i=1}^{N_{g}} \left(\sum_{j=1}^{N_{d}} P\left(i,j\right)\right)^{2}}{N_{z}}$$

4. Dependence non-uniformity:

$$DN=\frac{\sum_{j=1}^{N_{d}} \left(\sum_{i=1}^{N_{g}} P\left(i,j\right)\right)^{2}}{N_{z}}$$

5. Dependence non-uniformity normalized:

$$DNN=\frac{\sum_{j=1}^{N_{d}} \left(\sum_{i=1}^{N_{g}} P\left(i,j\right)\right)^{2}}{N_{z}^{2}}$$

6. Gray level variance:

$$GLV=\sum_{i=1}^{N_{g}} \sum_{j=1}^{N_{d}} p\left(i,j\right)\left(i-\mu\right)^{2}$$

7. Dependence variance:

$$DV=\sum_{i=1}^{N_{g}} \sum_{j=1}^{N_{d}} p\left(i,j\right)\left(j-\mu\right)^{2}$$

8. Dependence entropy:

$$DE=-\sum_{i=1}^{N_{g}} \sum_{j=1}^{N_{d}} p\left(i,j\right)\log_{2}\left(p\left(i,j\right)+\varepsilon\right)$$

9. Low gray level emphasis:

$$LGLE=\frac{\sum_{i=1}^{N_{g}} \sum_{j=1}^{N_{d}} \frac{P\left(i,j\right)}{i^{2}}}{N_{z}}$$

10. High gray level emphasis:

$$HGLE=\frac{\sum_{i=1}^{N_{g}} \sum_{j=1}^{N_{d}} P\left(i,j\right)i^{2}}{N_{z}}$$

11. Small dependence low gray level emphasis:

$$SDLGLE=\frac{\sum_{i=1}^{N_{g}} \sum_{j=1}^{N_{d}} \frac{P\left(i,j\right)}{i^{2}j^{2}}}{N_{z}}$$

12. Small dependence high gray level emphasis: Measures the joint distribution of small dependence with higher gray-level values.

13. Large dependence low gray level emphasis:

$$LDLGLE=\frac{\sum_{i=1}^{N_{g}} \sum_{j=1}^{N_{d}} \frac{P\left(i,j\right)j^{2}}{i^{2}}}{N_{z}}$$

14. Large dependence high gray level emphasis:

$$LDHGLE=\frac{\sum_{i=1}^{N_{g}} \sum_{j=1}^{N_{d}} P\left(i,j\right)i^{2}j^{2}}{N_{z}}$$

*Group 3: Shape features*

Shape features describe the two-dimensional size and shape of the ROI. These features are independent from the gray level intensity distribution in the ROI and are therefore only calculated on the non-derived image and mask.

Let:

- **N**_p_ represent the number of pixels included in the ROI.
- **N***_f_* represent the number of lines defining the circumference (perimeter) Mesh.
- **A** the surface area of the mesh in mm^2^.
- **P** the perimeter of the mesh in mm.

1. Mesh surface:

$$\begin{matrix} A_{i}=\frac{1}{2}Oa_{i}\times Ob_{i} \\ A=\sum_{i=1}^{N_{f}} A_{i} \end{matrix}$$

$O_{i}a_{i}$ and $O_{i}b_{i}$ are edges of the *i*^th^ triangle in the mesh, formed by vertices $a_{i}$, $b_{i}$ of the perimiter and the origin $O$.

2. Pixel surface:

$$A_{pixel}=\sum_{k=1}^{N_{v}} A_{k}$$

3. Perimeter:

$$\begin{matrix} P_{i}=\sqrt{\left(a_{i}-b_{i}\right)^{2}} \\ P=\sum_{i=1}^{N_{f}} P_{i} \end{matrix}$$

4. Perimeter to surface ratio:

$$perimeter to surface ratio=\frac{P}{A}$$

5. Sphericity:

$$sphericity=\frac{2\pi R}{P}=\frac{2\sqrt{\pi A}}{P}$$

6. Maximum 2D diameter:

$$spherical disproportion=\frac{P}{2\sqrt{\pi A}}$$

7. Major axis length:

$$major axis=4\sqrt{\lambda_{major}}$$

8. Minor axis length:

$$minor axis=4\sqrt{\lambda_{minor}}$$

9. Elongation:

$$elongation=\sqrt{\frac{\lambda_{minor}}{\lambda_{major}}}$$

*Group 4: Wavelet decompositions*

Wavelet-based features were derived from wavelet decompositions of the original ultrasound images using the “Coiflet 1” wavelet function. Each image was filtered using either a high band-pass filter (H) or low-band pass filter (L) in x and y directions, yielding 4 different combinations of decompositions. The wavelet decompositions of the original image X were labeled as X_LL_, X_LH_, X_HL_, and X_HH_.

*Group 5: Laplacian of Gaussian features*

Laplacian of Gaussian features were derived from the Laplacian of Gaussian image. A Laplacian of Gaussian image is obtained by convolving the image with the second derivative (Laplacian) of a Gaussian kernel. The width of the filter in the Gaussian kernel is determined by *σ* and can be used to emphasize more fine (low *σ* values) or coarse (high *σ* values) textures. We performed *σ =* {2, 3, 4, 5} during the feature extraction in this paper.

**Supplemental S2** Name of the extracted radiomics feature

| Radiomics Features |
| --- |
| original_shape2D_Elongation |
| log-sigma-2-0-mm-3D_firstorder_Kurtosis |
| log-sigma-2-0-mm-3D_glcm_JointEnergy |
| log-sigma-4-0-mm-3D_glcm_Idn |
| log-sigma-5-0-mm-3D_glszm_GrayLevelNonUniformity |
| log-sigma-5-0-mm-3D_glszm_ZoneEntropy |
| wavelet-LH_firstorder_Kurtosis |
| wavelet-LH_glszm_SmallAreaLowGrayLevelEmphasis |
| wavelet-LH_gldm_DependenceNonUniformityNormalized |
| wavelet-HL_glcm_SumSquares |
| wavelet-HH_firstorder_InterquartileRange |
| wavelet-HH_glcm_JointEntropy |
| wavelet-HH_glszm_SizeZoneNonUniformity |
| wavelet-LL_glrlm_LongRunHighGrayLevelEmphasis |

**Supplemental S3** Performance comparison of the deep learning algorithms in the training and test datasets

| Algorithm | Training | Test |  |  |  |  |
| --- | --- | --- | --- | --- | --- | --- |
|  | AUC | AUC | 95%CI | Accuracy | Sensitivity | Specificity |
| Resnet50 | 0.884 | 0.819 | 0.754-0.884 | 0.732 | 0.750 | 0.714 |
| Resnet34 | 0.683 | 0.661 | 0.599-0.722 | 0.618 | 0.696 | 0.540 |
| Resnet101 | 0.686 | 0.655 | 0.593-0.716 | 0.600 | 0.750 | 0.454 |
| Inception v3 | 0.634 | 0.633 | 0.571-0.696 | 0.590 | 0.743 | 0.441 |

Abbreviations: AUC, area under the curve; CI: confidence interval

**Supplemental S4** Structure of the ResNet50 used in the paper.

| name | output size | layer | parameter setting | number of blocks |
| --- | --- | --- | --- | --- |
| root | 32 × 32 | conv | k7, c64, s2, p3 | × 1 |
|  |  | pad | p1 |  |
|  |  | max pool | k3, s2, p0, d1 |  |
| block1 | 32 × 32 | group normalization1 | g32, c64 | × 3 |
|  |  | conv1 | k1, c64, s1 |  |
|  |  | group normalization2 | g32, c64 |  |
|  |  | conv2 | k3, c64, s1, p1 |  |
|  |  | group normalization3 | g32, c64 |  |
|  |  | conv3 | k1, c256, s1 |  |
|  |  | ReLU |  |  |
| block2 | 16 × 16 | group normalization1 | g32, c256 | × 4 |
|  |  | conv1 | k1, c128, s1 |  |
|  |  | group normalization2 | g32, c128 |  |
|  |  | conv2 | k3, c128, s2, p1 |  |
|  |  | group normalization3 | g32, c128 |  |
|  |  | conv3 | k1, c512, s1 |  |
|  |  | ReLU |  |  |
| block3 | 8 × 8 | group normalization1 | g32, c512 | × 6 |
|  |  | conv1 | k1, c256, s1 |  |
|  |  | group normalization2 | g32, c256 |  |
|  |  | conv2 | k3, c256, s2, p1 |  |
|  |  | group normalization3 | g32, c256 |  |
|  |  | conv3 | k1, c1024, s1 |  |
|  |  | ReLU |  |  |
| block4 | 4 × 4 | group normalization1 | g32, c1024 | × 3 |
|  |  | conv1 | k1, c512, s1 |  |
|  |  | group normalization2 | g32, c512 |  |
|  |  | conv2 | k3, c512, s2, p1 |  |
|  |  | group normalization3 | g32, c512 |  |
|  |  | conv3 | k1, c2048, s1 |  |
|  |  | ReLU |  |  |
| head | 1 × 1 | group normalization | g32, c2048 | × 1 |
|  |  | ReLU |  |  |
|  |  | average pool |  |  |
|  |  | conv | k1, c2, s1 |  |

k: kernel size, g: group size, s: stride, c: the number of channel, p: padding.

**Supplemental S5** Intra-operator ultrasound feature measurement consistency

| Ultrasound feature | Kappa |
| --- | --- |
| Size | 0.89 |
| Solid composition with hypoechoic echo | 0.85 |
| Tumour multifocality | 0.88 |
| Aspect ratio | 0.92 |
| Microcalcification | 0.83 |
| Tumour vascularity | 0.85 |
| Tumour location | 0.92 |
| Tumour position | 0.91 |
| Acoustic halo | 0.82 |
| ETE | 0.87 |
| Posterior acoustic attenuation | 0.90 |

Abbreviations: ETE, extrathyroidal extension
